# Role of tumor mutation burden-related signatures in the prognosis and immune microenvironment of pancreatic ductal adenocarcinoma

**DOI:** 10.1186/s12935-021-01900-4

**Published:** 2021-04-07

**Authors:** Rong Tang, Xiaomeng Liu, Wei Wang, Jie Hua, Jin Xu, Chen Liang, Qingcai Meng, Jiang Liu, Bo Zhang, Xianjun Yu, Si Shi

**Affiliations:** 1 Department of Pancreatic Surgery, Fudan University Shanghai Cancer Center, No. 270 Dong’An Road, Shanghai, 200032 China; 2 Department of Oncology, Shanghai Medical College, Fudan University, Shanghai, China; 3 Shanghai Pancreatic Cancer Institute, Shanghai, China; 4 Pancreatic Cancer Institute, Fudan University, Shanghai, China

**Keywords:** Pancreatic cancer, Tumor mutation burden, Microsatellite instability, Immune microenvironment, Molecular oncology

## Abstract

**Background:**

High tumor mutation burden (TMB) has gradually become a sensitive biomarker for predicting the response to immunotherapy in many cancers, including lung, bladder and head and neck cancers. However, whether high TMB predicts the response to immunotherapy and prognosis in pancreatic ductal adenocarcinoma (PDAC) remained obscure. Hence, it is significant to investigate the role of genes related to TMB (TRGs) in PDAC.

**Methods:**

The transcriptome and mutation data of PDAC was downloaded from The Cancer Genome Atlas-Pancreatic Adenocarcinoma (TCGA). Five independent external datasets of PDAC were chosen to validate parts of our results. qRT-PCR and immunohistochemical staining were also performed to promote the reliability of this study.

**Results:**

The median overall survival (OS) was significantly increased in TMB_low group compared with the counterpart with higher TMB score after tumor purity adjusted (*P* = 0.03). 718 differentially expressed TRGs were identified and functionally enriched in some oncogenic pathways. 67 TRGs were associated with OS in PDAC. A prognostic model for the OS was constructed and showed a high predictive accuracy (AUC = 0.849). We also found TMB score was associated with multiple immune components and signatures in tumor microenvironment. In addition, we identified a PDAC subgroup featured with TMB^low^Microsatellite instability^high^ (MSI^high^) was associated with prolonged OS and a key molecule, ANKRD55, potentially mediating the survival benefits.

**Conclusion:**

This study analyzed the biological function, prognosis value, implications for mutation landscape and potential influence on immune microenvironment of TRGs in PDAC, which contributed to get aware of the role of TMB in PDAC. Future studies are expected to investigate how these TRGs regulate the initiation, development or repression of PDAC.

**Supplementary Information:**

The online version contains supplementary material available at 10.1186/s12935-021-01900-4.

## Introduction

Pancreatic ductal adenocarcinoma (PDAC) is a lethal disease with a dismal prognosis [1]. The incidence and health burden of PDAC is increasing annually; however, effective treatment modalities are still extremely lacking [2]. The 5-year survival rate is only approximately 9% for patients with pancreatic cancer [3].

In recent years, anticancer immunotherapy has become an efficient method to curb tumor growth and metastasis in both the laboratory and the clinic [4, 5]. However, not all cancer types are suitable for immunotherapy given the low response rate observed in clinical trials [6, 7]. These kinds of tumors are called “immunotherapeutically cold tumors”, and PDAC is a typical cold tumor [8]. Immunosuppressive microenvironment was an important factor contributing to the form of cold tumor [8–10]. Lack of infiltration of anti-tumor lymphocytes and increased percentage of myeloid-derived suppressive cells are the main reasons for immunosuppressive microenvironment [11]. High tumor mutation burden (TMB) has gradually become a sensitive biomarker for predicting the response to immunotherapy in many cancers, including lung, bladder and head and neck cancers [12–16]. Nonetheless, a recent well-conducted study drew a different conclusion that high tumor mutation burden fails to predict immune checkpoint blockade response across all cancer types [17]. Moreover, whether high TMB could predict the response to immunotherapy in PDAC remains obscure [18, 19]. In this context, classifying PDAC patients based on a TMB score and comparing the difference in immune microenvironments and survival related to varied TMB scores contributes to evaluation of patients’ prognosis and possibility for acceptance of immunotherapy.

Other than TMB, many signatures could also be applied to predict the response to immunotherapy in patients with cancer [4, 5, 20–22]. For example, microsatellite instability (MSI), caused by a deficient DNA mismatch repair system, could identify good responders to immunotherapy in multiple cancers [23]. Alternatively, higher expression of PDL1 also predicted better efficacy of immunotherapy across many cancers [6, 24]. Investigating the correlations between TMB and these biomarkers as well as novel molecular classification schemes is also warranted.

In the present study, we investigated the role of TMB in the prognosis and immune microenvironment of PDAC patients. In addition, we developed a novel classification scheme based on TMB and MSI and identified molecules that potentially mediate the differences between the subtypes.

## Results

### DEGs between TMB_high and TMB_low PDAC patients

**Fig. 1:**
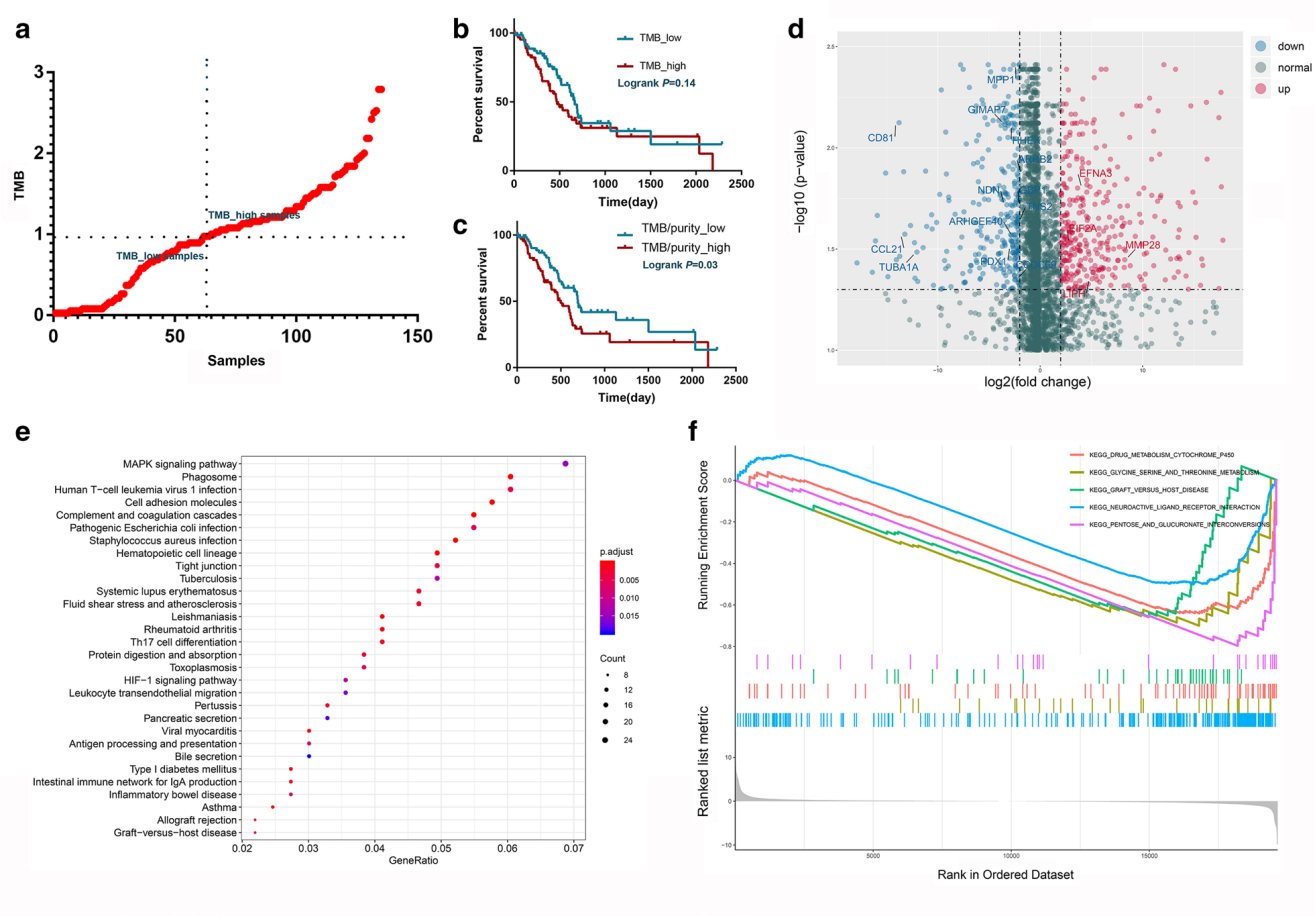
Survival comparison and differentially expressed genes between the TMB_high and TMB_low groups. **a** PDAC patients were divided into TMB_high and TMB_low groups based on the median TMB scores. **b** No obvious differences were detected between the TMB_high and TMB_low groups in terms of OS before tumor purity adjustment. **c** Patients with lower TMB had prolonged OS after adjustment for tumor purity. **d** A total of 718 differentially expressed genes were identified between the TMB_high and TMB_low groups (LogFC > 2 and FDR < 0.05). **e** KEGG analysis revealed the potential functions of the differentially expressed genes. Gene ratio refers to the percentage of genes of a specific pathway in TRGs. The size of round symbols refers to the counts of enriched genes. **f** GSEA revealed the potential functions of the differentially expressed genes

Originally, the transcriptome data of 186 patients were downloaded from TCGA-PAAD. A total of 150 PDAC patients were included after 32 patients with other pancreatic neoplasms were excluded. Among these PDAC patients, 136 patients had TMB data and hence were enrolled in the following analysis. In addition, one patient whose TMB score deviated extremely from that of other patients was also removed from the present study. The patients were further divided into two groups (TMB_high and TMB_low) based on the median value of TMB across the whole cohort (Fig. 1a). First, we compared the baseline characteristics between the two groups. The proportion of tumors in the pancreatic head was larger in the TMB_low group (*P* = 0.007), while tumor purity was increased in the TMB_high group (*P* < 0.0001) (Table 1). Second, we investigated whether the TMB score was associated with the prognosis of PDAC patients. The results showed no significant difference between the two groups, although the median OS was slightly prolonged in patients with lower TMB scores (Fig. 1b; *P* = 0.14). However, TMB was to some extent determined by tumor purity. Given the obvious difference in baseline tumor purity we observed between the two groups, we compared OS between the TMB_high and TMB_low groups after adjusting for tumor purity. Interestingly, the median OS was significantly increased in the TMB_low group, which had adjusted tumor purity, compared with the TMB_high group (Fig. 1c; *P* = 0.03). Third, we identified TRGs and their potential functions. With strict screening criteria, a total of 718 DEGs were identified (LogFC > 2 and FDR < 0.05; Fig. 1d).

**Table 1:** The baseline characteristics of PDAC patients included in this study

	TMB_low (n = 67)	TMB_high (n = 68)	
Age (year)^a^	66.22 ± 1.33	63.82 ± 1.40	p = 0.22
Gender (female %)	32 (49.2%)	27 (40.3%)	p = 0.38
Location (head %)	59 (88.1%)	46 (67.7%)	p = 0.007*
T3T4 (%)	56 (83.6%)	61 (89.7%)	p = 0.32
N1 (%)	55 (80.9%)	44 (66.7%)	p = 0.07
AJCC pathologic tumor stage (a2 ~)	56 (83.6%)	46 (68.7%)	p = 0.07
KRAS	61 (91%)	64 (94.1%)	p = 0.53
Tumor purity^a^	0.27 ± 0.02	0.47 ± 0.02	p < 0.0001*

^*^Statistically significant

^a^Data was presented as mean ± deviance

The KEGG analysis revealed that the TRGs were enriched in some classic oncologic signaling pathways, such as MAPK and HIF-1 signaling. In addition, we also observed that these TRGs may be involved in some pancreatic physiopathologies, such as pancreatic secretion and diabetes. TMB is a common biomarker for predicting the response to immunotherapy in multiple cancers. Here, we showed that some TRGs may regulate the remodeling of the immune microenvironment in PDAC. For example, TRGs may affect Th17 cell differentiation, leukocyte transendothelial migration, antigen presentation and processing and IgA production (Fig. 1e). The GO analysis also demonstrated that the TRGs were involved in neutrophil-mediated immunity, negative regulation of immune system process and MHC class II protein complex (Additional file 1: Figure S1A). Furthermore, we performed GSEA to identify the upregulation or downregulation of certain gene sets in groups with different TMB scores, and the top five enriched gene sets in two gene lists (KEGG and GO) were visualized. According to the GSEA results, metabolic remodeling might be a potential downstream factor for TMB variation, since terms such as drug metabolism, cytochrome P450, glycine, serine and threonine metabolism, pentose and glucuronate interconversions and hormone regulation were enriched in PDAC patients with decreased TMB scores (Fig. 1f and Additional file 1: Figure S1B).

**Fig. 2:**
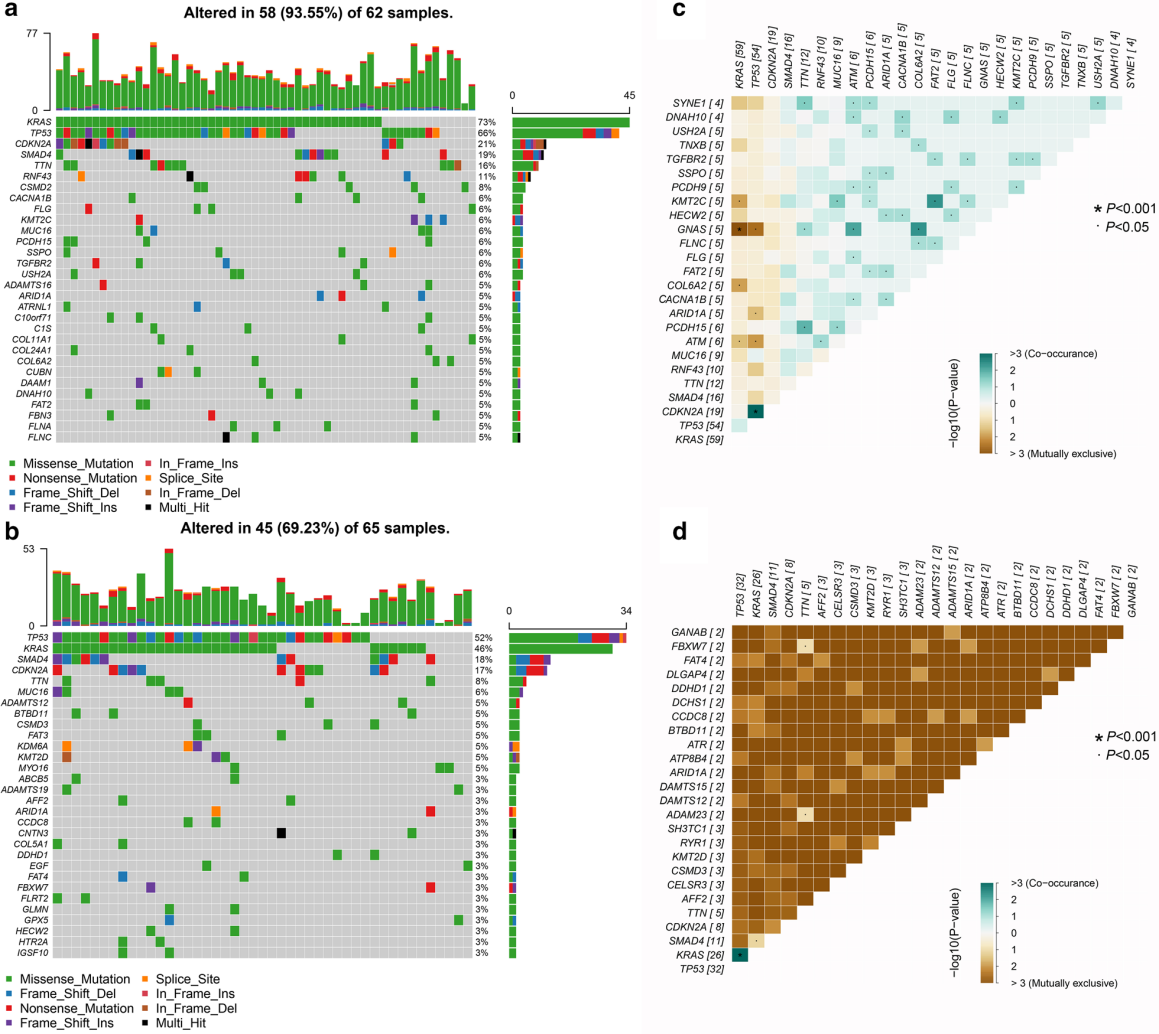
The mutation landscape of patients with high and low TMB scores. **a**, **b** Waterfall plot visualizing the top 30 genes that mutated most frequently in the TMB_high and TMB_low groups, respectively. The mutation percentage refers to the ratio of samples harbored the mutations in the 30 genes in all samples. **c**, **d** The co-occurrence and mutual exclusion of mutated genes in the TMB_high and TMB_low groups, respectively

Next, we compared the most frequent somatic mutations between the two groups. Overall, four driver genes, TP53, KRAS, SMAD4 and CDKN2A, were similar between the TMB_high and TMB_low cohorts in terms of their mutation frequencies. The ranking of other genes showed slight changes, as shown in Fig. 2a, b. For example, the mutation frequency of DAMTS15 was ranked 10th in the TMB_high group (3%), but it dropped out of the top 20 most frequently mutated genes. In addition, the co-occurrence and mutual exclusion between mutated genes were significantly different between the TMB_high and TMB_low groups. In TMB_high samples, the co-occurrence between mutated genes was extremely common, while mutual exclusion was observed for only the KRAS-KMT2C, KRAS-GNAS, KRAS-COL6A2, KRAS-ATM, TP53-GNAS, TP53-ARID1A and TP53-ATM pairs (Fig. 2c). In contrast, in TMB-low samples, less co-occurrence and mutual exclusion were observed, and a common trend of mutual exclusion widely existed in this cohort (Fig. 2d). In addition, we visualized the mutational landscape of the two groups in Additional file 1: Figure S2.

### The correlation between TRG expression and the prognosis of PDAC patients

**Fig. 3:**
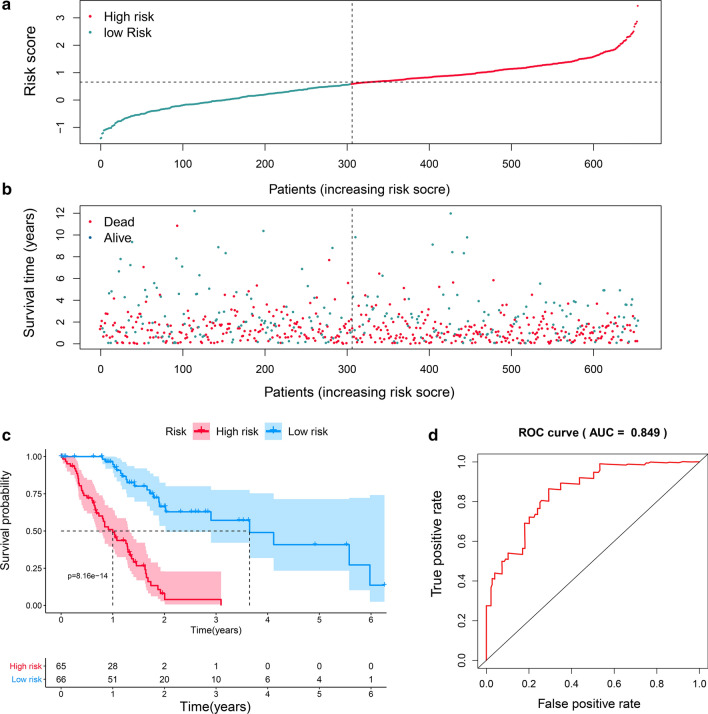
A prognostic model based on TRGs for predicting the OS of PDAC patients. **a** PDAC patients were divided into high- and low-risk groups based on their score calculated by the model via lasso regression. **b** The survival time and status of PDAC patients varied with increasing risk scores. **c** Patients with lower lasso risk scores showed prolonged OS. **d** ROC curve demonstrated a high accuracy of the constructed model (AUC = 0.849).

We conducted univariate Cox regression to identify survival-related TRGs in PDAC patients. The expression of 9.3% (67/718) of TRGs was associated with OS, where 34 genes were favorable survival factors, while the other 33 genes were unfavorable survival factors (Additional file 1: Table S2). Given the capability of these TRGs to predict the OS of PDAC, we then constructed a prognostic model based on their expression levels using lasso regression (Additional file 1: Figure S3). Fifty-one genes were removed after lasso regression to avoid the overfitting phenomenon. Finally, 16 genes were retained for subsequent model construction. The coefficient of each gene in the model is provided in Additional file 1: Table S3. The patients were divided into high- and low-risk groups based on their risk score calculated by the model (Fig. 3a). Their survival time and status varied along with an increased risk score (Fig. 3b). OS was significantly prolonged in the low-risk group (Fig. 3c). ROC curves were calculated to evaluate the accuracy of the model. The area under the curve (AUC) of this model was 0.849, which demonstrated good accuracy in predicting the OS of PDAC patients (Fig. 3d). Then, we created a validation cohort consisting of 655 PDAC patients from five independent datasets (Additional file 1: Table S4). Using the same genes, coefficients and cutoff values, we divided the patients into high- and low-risk groups (N = 350 and 305, respectively; Additional file 1: Figure S4A-B). The survival analysis showed that our model could accurately distinguish patients with dismal prognosis from the whole cohort (*P* = 0.03) (Additional file 1: Figure S4C-E).

To further decipher the role of the genes used in the prognostic model, we investigated the differential expression of these genes between tumor and normal samples using bulk sequencing data from the TCGA and Genotype-Tissue Expression (GTEx) databases. Among them, four genes were upregulated in tumor samples and associated with dismal prognosis (Additional file 1: Figure S5). Given the cell heterogeneity in tumor tissues, the differential expression of specific genes may not be caused by tumor cells themselves. Hence, we confirmed the differential expression of genes in a pancreatic cancer cell line and a normal pancreatic ductal cell line. The relative mRNA levels of these genes in the cell lines showed a similar trend as the bulk sequencing results, except no difference was found in terms of the mRNA expression of MMP28 between the Capan-1 cell line and the HPDE cell line (Additional file 1: Figure S5).

### The TMB score is associated with the remodeling of the immune microenvironment in PDAC

**Fig. 4:**
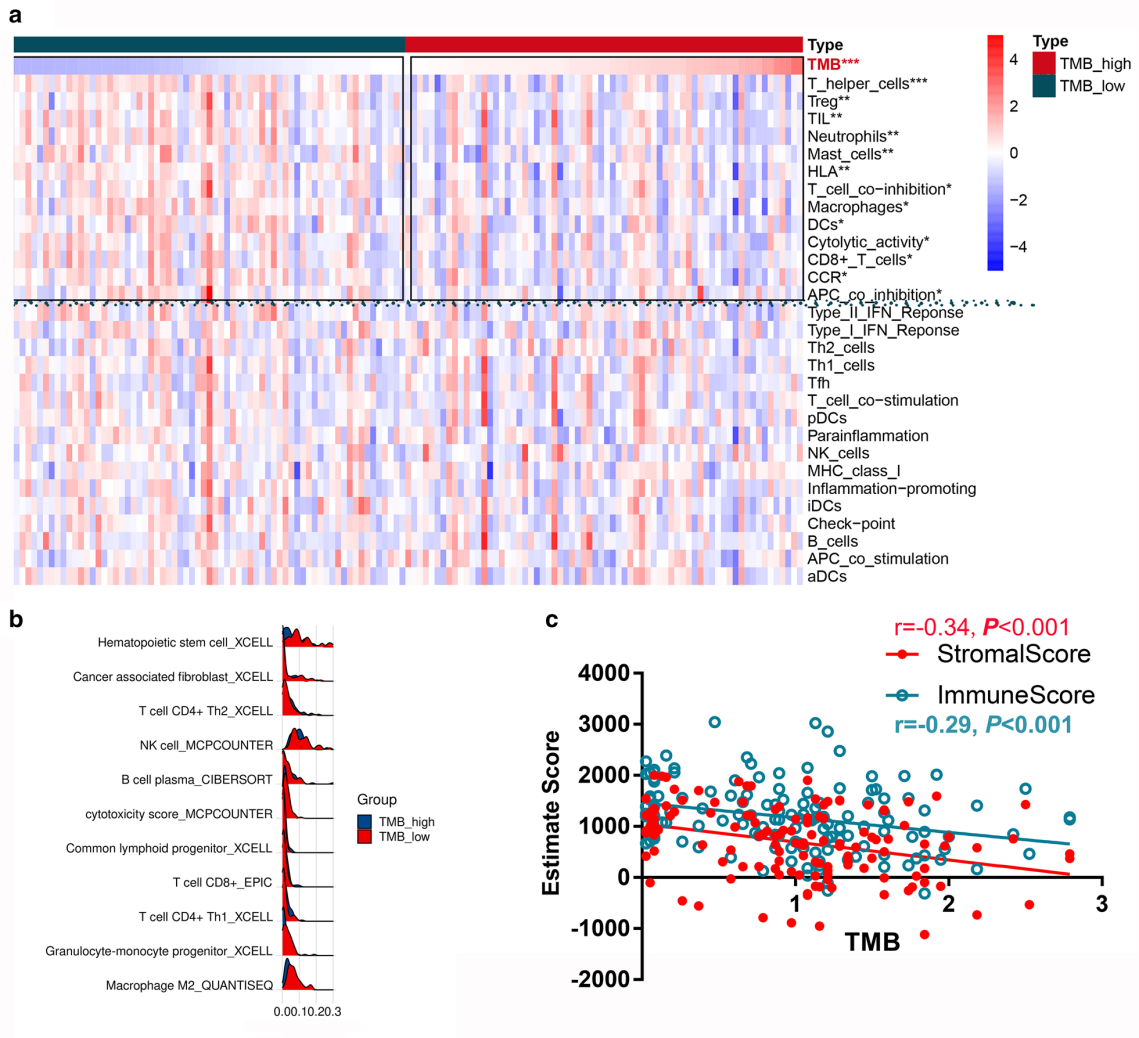
The correlation between the TMB score and the immune microenvironment in PDAC. **a** The differences between the TMB_high and TMB_low groups in terms of 29 immune signatures. **b** Partial presentation of the correlation between the TMB scores and infiltrated immune cells using different algorithms. **c** The TMB score is negatively associated with the immune and stromal scores (*P* < 0.001)

Although TMB is regarded as an effective biomarker for predicting the response to immunotherapy in patients with solid tumors, the effectiveness of TMB in some immunologically cold tumors, such as PDAC, remains controversial. In this context, we analyzed the association between the TMB score and immune cell infiltration using multiple algorithms. First, we used ssGSEA to calculate the activity of 29 immune signatures and further analyzed their correlation with TMB (Fig. 4a). The results showed that TMB was negatively associated with many anticancer signatures, such as CD8+ T cells and cytolytic activity. However, TMB was also negatively correlated with some immunoinhibitory factors, such as Treg cells and APC co-inhibition. Of note, different algorithms for the estimation of immune infiltration may yield conflicting conclusions. For example, while TMB was negatively associated with the fraction of CD8+ T cells using ssGSEA, Timer and CIBERSORT (Additional file 1: Table S5), we found a positive correlation between CD8+ T cells and the TMB score using the EPIC algorithm (Fig. 4b). Some results also seemed to be complex and conflicting. For instance, more cancer-associated fibroblasts, which are normally seen as protumoral factors, were infiltrated in the TMB_low group. However, several anticancer factors, such as NK cells and cytotoxic scores, were also enriched in the TMB_low group (Fig. 4b). Negative correlations were observed between TMB and stromal (r = − 0.34, *P* < 0.001) and immune scores (r = − 0.29, *P* < 0.001) (Fig. 4c). Overall, the TMB score was associated with multiple components in the tumor microenvironment; however, whether it is an effective biomarker reflecting anticancer immunity remains obscure in PDAC in view of the complex relationship between TMB and various immune signatures. Under this circumstance, we performed a more precise PDAC classification and focused on single gene-level regulation that mediated the influence of TMB on PDAC development in the following analysis.

### ***A PDAC subgroup featuring TMB***^***low***^***MSI***^***high***^*** was associated with prolonged OS***

**Fig. 5:**
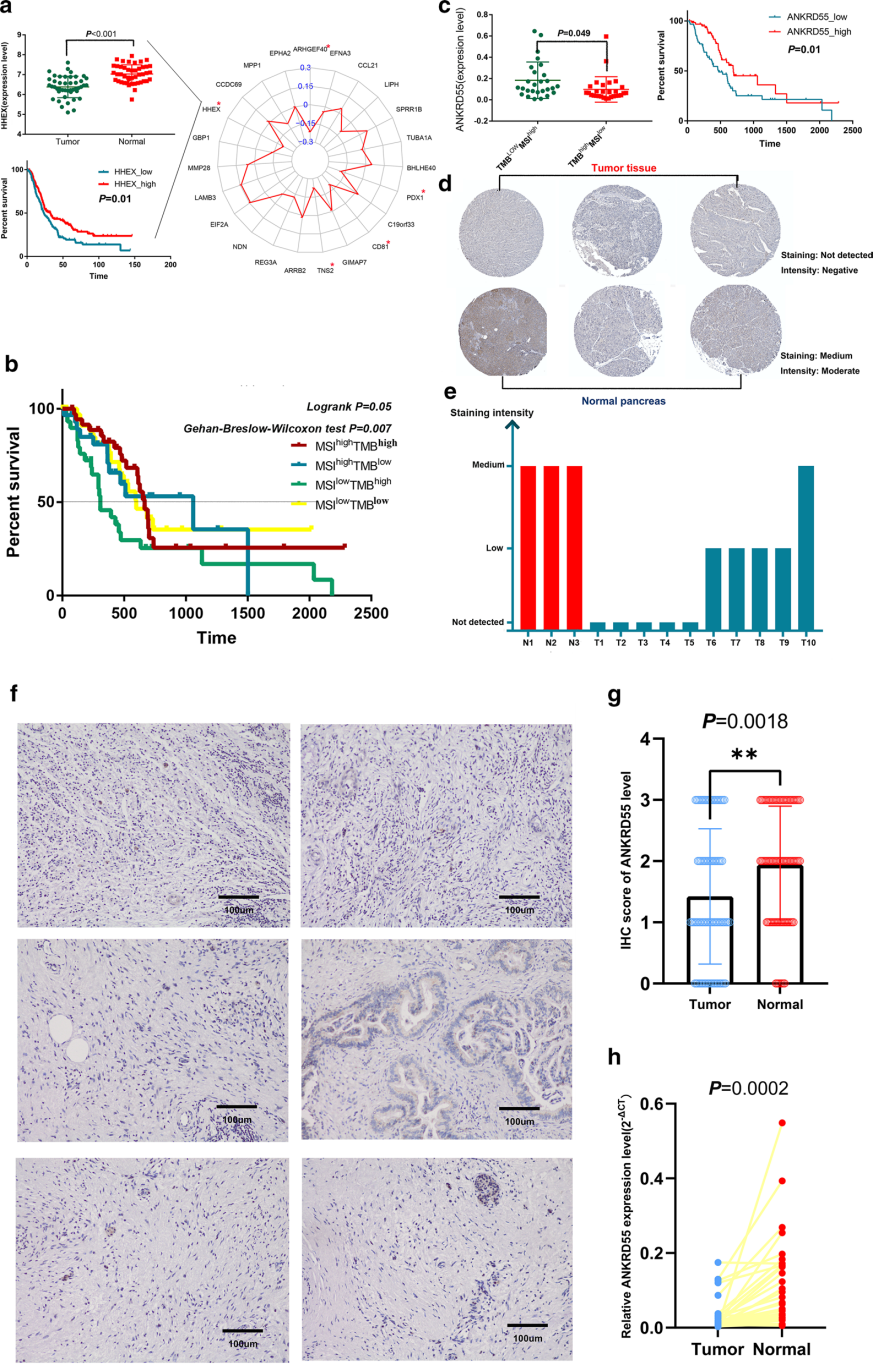
A subgroup featuring TMB^low^MSI^high^ showed prolonged OS. **a** The correlation between survival-related TRGs and MSI. HHEX was downregulated in tumor tissues (GSE28735) and positively associated with prolonged OS (E-MTAB-6134). **b** The OS of patients with TMB^low^MSI^high^ was significantly increased compared with that of other subtypes (*P* < 0.05). **c** ANKRD55 is significantly overexpressed in the TMB^low^MSI^high^ subtype and positively associated with prolonged OS. **d**, **e** ANKRD55 is universally upregulated in PDAC tissues compared with normal pancreatic tissues using immunohistochemical staining. **f** ANKRD55 is overexpressed in the stroma and morphologically normal pancreatic ductal structures. **g** The IHC scores of ANKRD55 is significantly decreased in PDAC samples compared with normal adjacent tissues (*P* = 0.0018). **h** The mRNA level of ANKRD55 is significantly downregulated in PDAC samples compared with normal adjacent tissues (*P* = 0.0002)

Previous studies have indicated that cancers with high MSI respond very well to immune checkpoint inhibitors [25, 26]. We hence explored the correlation between survival-related TRGs and MSI. Five genes were found to be negatively associated with MSI (Fig. 5a). Meanwhile, all these genes were upregulated in tumor samples with lower TMB. Among the 5 genes, PDX1 is a classical negative regulator of PDAC initiation, as shown in a PDX1-deleted PDAC animal model, but the roles of the other genes in PDAC remain unclear. Then, we confirmed their differential expression between tumor and normal tissues and survival relevance in the validation cohort. HHEX was identified as a gene of interest because it was downregulated in tumor tissues and was associated with prolonged OS (Fig. 5a).

To obtain more information on the influence of TMB and MSI on PDAC survival, we divided the patients into four groups based on the median TMB and MSI scores and then compared the OS among the subtypes. We found that the TMB^low^MSI^high^ group had the longest OS with marginal statistical significance (log-rank test *P* = 0.05; Gehan-Breslow-Wilcoxon test *P* = 0.007; Fig. 5b). Interestingly, the TMB^high^MSI^low^ subtype had the shortest OS. Hence, we analyzed the DEGs between the TMB^low^MSI^high^ and TMB^high^MSI^low^ groups. A total of 14 genes were deemed to be differentially expressed, and only one of them (ANKRD55) was associated with patient OS (Fig. 5c). In this context, we raised the possibility that ANKRD55 mediated the survival benefits of the TMB^low^MSI^high^ subtype. Therefore, we further studied whether ANKRD55 was differentially expressed between tumor and normal tissues. Immunohistochemical staining demonstrated that ANKRD55 was universally downregulated or even not expressed in PDAC samples. In contrast, it had medium expression across normal pancreatic tissues (Fig. 5d, e). To further validate our findings, we detected the expression level of ANKRD55 in patients from our center. Overall, ANKRD55 was highly expressed in stromal and normal ductal structures but rarely expressed in malignant ductal structures, which is consistent with the HPA results.

**Fig. 6:**
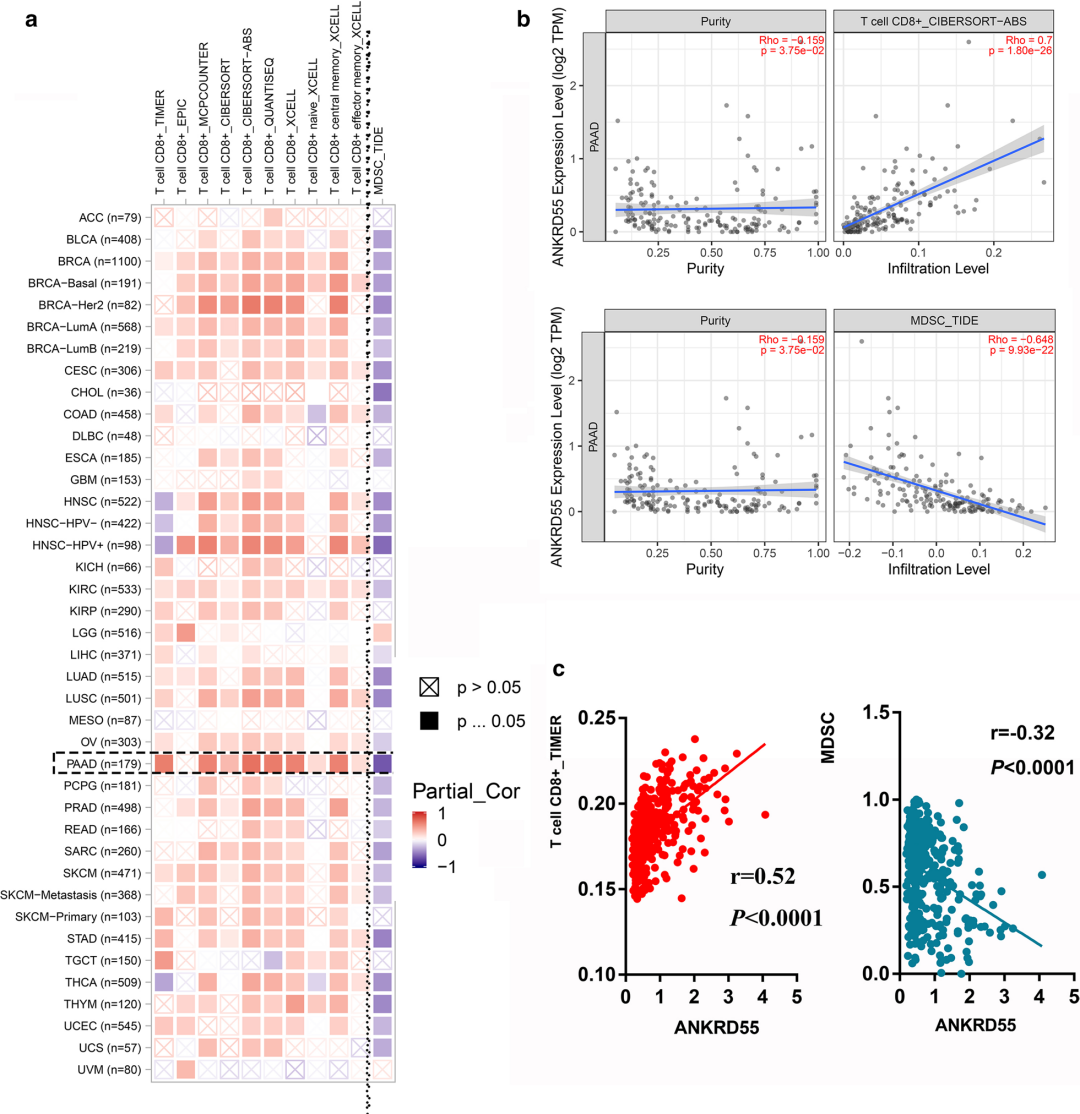
The expression of ANKRD55 is associated with a higher infiltration of CD8+ T cells and a lower infiltration of MDSCs. **a** ANKRD55 is associated with a higher infiltration of CD8+ T cells and a lower infiltration of MDSCs in most cancers. **b** ANKRD55 expression is positively associated with CD8+ T cells and negatively associated with MDSCs in pancreatic cancer based on TCGA dataset. **c** ANKRD55 expression is positively associated with CD8+ T cells and negatively associated with MDSCs in pancreatic cancer based on GSE71729

Next, we sought to determine whether ANKRD55 affected the survival of PDAC patients through immune regulation. Interestingly, ANKRD55 expression was positively correlated with CD8+ T cell infiltration not only in PDAC (r = 0.70, *P* < 0.001) but also in most other tumors (Fig. 6a, b). In addition, its expression was negatively associated with myeloid-derived suppressor cell (MDSC) infiltration (r = − 0.65, *P* < 0.001; Fig. 6c). Then, we confirmed this association in the GEO cohort (Fig. 6c). Therefore, it is plausible that ANKRD55 inhibited PDAC development through CD8+ T cell enrichment and MDSC exclusion. We further explored the relationships between ANKRD55 expression and immune checkpoints, DNA repair-related genes and DNA transmethylase in the pan-cancer profile. The results showed that ANKRD55 expression was positively associated with most immune checkpoints in cancers, suggesting that although ANKRD55 predicted more intratumorally infiltrated CD8+ T cells, cancer cells may still evade immune system-mediated killing through immune checkpoint overexpression (Additional file 1: Figure S6A). Among the four DNA transmethylases, only DNMT1 and DNMT2 were associated with ANKRD55 expression in pancreatic cancer (Additional file 1: Figure S6B). Additionally, MLH1 was positively associated with ANKRD55 expression, while EPCAM was negatively associated with ANKRD55 expression in pancreatic cancer (Additional file 1: Figure S6C).

## Discussion

Many achievements have been made in the immunotherapy of cancers. However, not all patients benefit from immunotherapy [27, 28]. High TMB are sensitive biomarkers for screening good responders to immunotherapy and have been shown to be more significantly associated with the response to PD1 and PD-L1 blockade than PD-1 or PD-L1 expression [29]. Mechanistically, high TMB provides more opportunities for “non-self” neoantigen production, which activates the enrichment of immune cells [29]. Nonetheless, such theories were confirmed in only some immunotherapeutically hot tumors, while in cold tumors such as PDAC, such rules may not be applicable.

Many clinical trials have explored the value of immunotherapy in PDAC. Most of these studies reported an extremely low response rate to immunotherapy in PDAC patients, especially for those who received single immune checkpoint-based treatment [30–33]. Some plausible reasons may account for the difficulty in curing PDAC using immunological methods. On the one hand, intratumoral hypoxia in PDAC is a predominant driver of the recruitment of immunosuppressive cells through cancer-associated fibroblast activation [34]. On the other hand, pancreatic cancer has a low mutation load compared to other solid tumors, which partially restrains the production of neoantigens that induce an effective immune response [35]. To better understand the dilemma in immunotherapy for PDAC, we investigated how TMB influences the prognosis and immune microenvironment of PDAC in the present study.

We found that TMB was negatively associated with the OS of PDAC patients after adjusting for tumor purity. This suggests that high TMB could be a predictor for the prognosis of patients with PDAC beyond its conventional role in patients’ selection for immunotherapy. Hence, we further investigated whether the TMB score impacted immune cell infiltration in PDAC. By analyzing the activity of 29 immune signatures in groups with different TMB scores, we found a complicated phenomenon in which although some tumor-inhibitory cells were enriched in the TMB_low group, some protumoral cells, such as cancer-associated fibroblasts or Tregs, were also enriched in this group. Given that MSI is also a biomarker for immunotherapeutic response [36, 37], we established two new PDAC subtypes based on the median value of the TMB and MSI scores. Interestingly, the TMB^low^MSI^high^ group featured significantly prolonged OS compared with their counterparts. Furthermore, we found that ANKRD55 was overexpressed in the TMB^low^MSI^high^ group and positively associated with the OS of PDAC. Immunohistochemical staining and qPCR indicated that this gene was downregulated in tumor tissues. Notably, the expression of ANKRD55 was significantly associated with higher infiltration of CD8+ T cells and lower infiltration of MDSCs, which suggested that this gene may mediate the survival benefits observed in the TMB^low^MSI^high^ group through the remodeling of the immune microenvironment. Previous studies have reported that single nucleotide polymorphisms in ANKRD55, an autoimmune risk protein [38, 39], are associated with type 2 diabetes susceptibility [40]. Interestingly, type 2 diabetes is an important risk factor for PDAC development and progression [41].

The prognostic model we generated showed high accuracy both in training dataset and validation cohorts. Recently, a lot of studies constructed prognostic model based on transcriptome data for pancreatic cancer [42–46]. Compared these models, our model showed comparable accuracy and premium stability based on large sample size. Besides, the presents study is the first to establish prognostic model based on TRGs.

Certainly, this study has several limitations to consider. First, the TRGs were identified using only TCGA data because other datasets could not provide relevant exon sequencing data to compute TMB. Second, although we systematically investigated the prognostic implications and immune microenvironment of TRGs in pancreatic cancer, we did not present direct evidence about whether and how TRGs regulate the response to immunotherapy in PDAC, which was limited due to the inaccessibility of resected samples previously exposed to immunotherapy clinically. The present study also has some strengths. First, this is the first study to systematically determine the role of TRGs in the prognosis and immune microenvironment of PDAC. Second, we classified PDAC samples into different subtypes with various OS outcomes based on TMB and MSI and identified a potential molecule that may mediate the observed survival benefits. Third, in addition to in silico bioinformatic analysis, we performed qRT-PCR and immunohistochemical staining with human samples to validate parts of our results.

In conclusion, this study analyzed the biological functions, prognostic value, implications for the mutational landscape and potential influence on the immune microenvironment of TRGs in PDAC, which contributed to increasing the awareness of the role of TMB in PDAC. Future studies are expected to investigate how these TRGs regulate the initiation, development or repression of PDAC.

## Materials and methods

### Data source and selection

RNA-sequencing data, including read counts and fragments per kilobase per million (FPKM), were collected from The Cancer Genome Atlas (TCGA)-Pancreatic Adenocarcinoma (PAAD) dataset [47]. According to the annotation of TCGA-PAAD, we excluded nonductal-derived tumors and normal adjacent samples. Only PDAC samples remained for subsequent bioinformatic analysis. In addition, microarray gene expression data from E-MTAB-6134, GSE21501, GSE57495, GSE85916 and GSE71729 were downloaded and analyzed as the validation cohort. Clinical data such as overall survival (OS) were also downloaded from the abovementioned datasets.

### Classification of tumor samples based on the TMB score

TMB is a measure of the total number of mutations per megabyte of tumor tissue. We calculated TMB using the “maftools” R package (version 2.2). The patients were divided into two groups (TMB_high and TMB_low) based on the median value of TMB across the whole population. The differentially expressed genes (DEGs) between the TMB_high and TMB_low groups were regarded as TMB-related genes (TRGs). The Wilcoxon test was used to detect the differences in gene expression with the “limma” R package (version 3.4). The cutoff values to define the DEGs were log(fold change (FC)) > 2 and false discovery rate (FDR) < 0.05. Gene Ontology (GO) functional enrichment analysis and KOBAS-Kyoto Encyclopedia of Genes and Genomes (KEGG) pathway analysis of DEGs were performed by the “clusterProfiler”, “org.Hs.eg.db”, “plot”, and “ggplot2” R packages. Gene set enrichment analysis (GSEA) was also performed to explore the functions of the TRGs using the “clusterProfiler”, “org.Hs.eg.db”, “enrichplot” and “limma” R packages. A waterfall plot was constructed to visualize the top 20 genes that mutated most frequently in the two groups.

### The clinical relevance of TRG expression levels and the construction of a prognostic model

First, univariate Cox regression analysis was conducted to screen the TRGs that were significantly associated with the prognosis of PDAC (P < 0.05). Then, least absolute shrinkage and selection operator (lasso) regression was performed to calculate the risk coefficient of each gene after the removal of some genes with a risk of overfitting according to the partial likelihood deviance and lambda value (the lambda value is determined by the smallest likelihood deviance; the coefficient-lambda curve demonstrates the genes that are eligible when the lambda value is determined) (glmnet, version 2.0–18). We calculated the risk score for each patient using the following formula: Lasso risk = ${\sum }_{i=1}^{n}Coef\times xi.$ Finally, the remaining genes were utilized to construct a predictive model for the prognosis of PDAC. The samples with the top 50% risk value were regarded as “high risk”, while the samples with the bottom 50% risk value were regarded as “low risk”. Kaplan–Meier analysis was performed to compare the difference in OS between TMB_high and TMB_low patients. A receiver operating characteristic (ROC) curve was generated to assess the predictive value of the constructed model using the “survivalROC” package. A validation cohort consisting of the E-MTAB-6134 and 4 Gene Expression Omnibus (GEO) datasets was used to confirm the accuracy of the model. We adjusted for the expression levels of genes in different datasets, which ensured optimized comparability between the validation cohort and the TCGA cohort. First, we standardized each gene’s expression level according to the following formula: ${x}_{std}=\frac{{x}_{i}-\stackrel{-}{x}}{s}$, $\stackrel{-}{x}$=$\frac{1}{n}\sum_{i=1}^{n}{x}_{i}$, s = $\sqrt{\frac{1}{n-1}\sum_{i=1}^{n}{({x}_{i}-\stackrel{-}{x})}^{2}}$. Then, we adjusted each ${X}_{std}$ to match the training data of TCGA by the following formula:

$$ x_{adj} = x_{std} \times s_{train} + \overline{x}_{train} $$

### Cell cultures and qRT-PCR

The human pancreatic cancer cell line Capan-1, Panc-1, SW1990 and Mia-Paca2 were obtained from the American Type Culture Collection. Capan-1 cells were cultured in Iscove's modified Dulbecco’s medium (IMDM) with 10% fetal bovine serum. Panc-1, SW1990 and Mia-Paca2 were cultured in dulbecco’s modified eagle medium (DMEM). 43 pairs of resected pancreatic cancer tissues and adjacent normal tissues preserved in RNA later. Then, RNA was extracted from tissues using SteadyPure Universal RNA Extraction Kit (AG21017). Quantitative real-time PCR was performed as described previously [48]. All reactions were run in triplicate. The primer sequences are listed as Additional file 1: Table S1.

### Immunohistochemical staining

85 clinical tissue samples used in this study for immunohistochemical staining were obtained from patients diagnosed with pancreatic cancer at Fudan University Shanghai Cancer Center. Prior patient consent and approval from the Institutional Research Ethics Committee were obtained. Immunohistochemical staining of paraffin-embedded tissues with antibodies against ANKRD55 was performed to detect the expression of ANKRD55 according to standard immunohistochemical procedures [48]. Anti-ANKRD55 antibody (NBP2-14719, Novus) was used at a dilution factor of 1:100. The staining intensity of ANKRD55 were scored as 0 (negative), 1 (weak), 2 (moderate) and 3 (strong).

### The relationship between TRGs and the immune microenvironment

We used two methods to estimate the fraction of immunity-related components in the tumor microenvironment. First, single-sample gene set enrichment analysis (ssGSEA) was conducted based on the expression levels of 29 immunity-associated signatures using the “GSEABase” R package (version 1.4). Second, we assessed the infiltration of immune cells with Tumor Immune Estimation Resource (TIMER) 2.0 (https://cistrome.shinyapps.io/timer/), where six algorithms, comprising TIMER, CIBERSORT, quanTIseq, xCell, MCP-counter and EPIC, were applied in the analysis. The “estimate” R package (version 1.0) was used to calculate immune and stromal scores. The quantitative correlation between TRG expression and immune infiltration was evaluated using the Pearson correlation coefficient (r).

### Combining MSI with TMB to determine PDAC subtypes

Given that MSI is also a biomarker for the response to immunotherapy in solid tumors, we divided the patients into four subgroups (TMB^high^MSI^high^, TMB^high^MSI^low^, TMB^low^MSI^low^ and TMB^low^MSI^high^) based on the median TMB/MSI scores. The MSI scores of each PDAC sample were derived from a previous study [49]. Kaplan–Meier curves were constructed to compare the OS among these groups. Next, we explored the association between TRG expression and the MSI score. TRGs that significantly correlate with MSI were further investigated in terms of their differential expression between tumor and normal adjacent tissues and their survival relevance. We further explored the DEGs between TMB^high^MSI^low^ and TMB^low^MSI^high^ patients using the Wilcoxon test and then explored the correlation of these genes with patient survival (R packages: “survival”, version 3.18; “survminer”, version 0.4.6). Immunohistochemical staining of the genes of interest was investigated in The Human Protein Atlas database (https://www.proteinatlas.org/). Only the samples stained by the same antibody were included in our analysis. We also used TIMER 2.0 to explore the associations between the gene of interest and CD8+ T cell infiltration and myeloid-derived suppressor cells after adjusting for tumor purity.

## Supplementary Information

**Additional file 1: Figure S1.** GO analysis and GSEA of the TRGs. (A) GO analysis showed the function of TRGs. (B) GSEA showed differentially enriched pathways of TRGs using the GO gene-sets. **Figure S2.** Mutational landscape of the TMB_high and TMB_low groups. **Figure S3.** Lasso regression identifies 16 TRGs for model construction. (A) The curve shows that the partial likelihood deviance changed along with the lambda value. The lambda value is determined when the partial likelihood deviance is at its minimum value. (B) When the lambda value is determined, the corresponding coefficient of each gene can be determined. **Figure S4.** Validation of the constructed model using the same coefficients and cutoff values. (A) PDAC patients were divided into high- and low-risk groups based on their scores calculated by the model. (B) The survival time and status of PDAC patients varied with increasing risk scores. (C) Patients with low lasso risk scores had prolonged OS. (D) Patients with high lasso risk scores featured increased cumulative hazards. (E) ROC curve to assess the accuracy of the model. **Figure S5.** The differential expression of four genes in the prognostic model based on bulk sequencing and qPCR validation. Target genes from left to right were GBP1, HIST1H1C, MMP28 and PPP1R15A, respectively. Cell-lines from top to bottom were capan-1, panc-1, Mia-paca-2 and SW1990. **Figure S6.** Correlation analysis between ANKRD55 expression and immune checkpoints (A) DNA transmethylases (B) and DNA repair-related proteins (C). **Table S1.** Primer sequencing of the genes that need to be validate by PCR. **Table S2.** A summary of survival-associated TRGs in PDAC. **Table S3.** Genes used in the construction of prognosis model. **Table S4.** Characterization of validation cohorts. **Table S5.** The association between CD8 + T cells infiltration and TMB score.

## Data Availability

The datasets generated analyzed during the current study are available in the TCGA repository (https://www.cancer.gov/about-nci/organization/ccg/research/structural-genomics/tcga); GEO datasets (https://www.ncbi.nlm.nih.gov/gds/); ArrayExpress (https://www.ebi.ac.uk/arrayexpress/).
